# A Clinically Oriented antimicrobial Resistance surveillance Network (ACORN): pilot implementation in three countries in Southeast Asia, 2019-2020

**DOI:** 10.12688/wellcomeopenres.18317.1

**Published:** 2022-12-22

**Authors:** H. Rogier van Doorn, Thyl Miliya, Anousone Douangnouvong, Ngan Ta Thi Dieu, Chansovannara Soputhy, Meymey Lem, Danoy Chommanam, Valy Keoluangkhot, Bandith Soumphonphakdy, Khaysy Rassavong, Khamphong Thanadabouth, Manoloth Sayarath, Vilada Chansamouth, Minh Dien Vu, Phu Khiem Dong, Van Duong Dang, Van Bac Tran, Thi Kim Yen Do, Thi Ngoc Ninh, Hong Long Nguyen, Ngoc Hao Kim, Sothea Prak, Manivanh Vongsouvath, Dinh Trang Van, Thi Kim Tuyen Nguyen, Hong Khanh Nguyen, Raph L. Hamers, Clare Ling, Tamalee Roberts, Naomi Waithira, Prapass Wannapinij, Tien Viet Dung Vu, Olivier Celhay, Chanpheaktra Ngoun, Susath Vongphachanh, Ngoc Thach Pham, Elizabeth A. Ashley, Paul Turner

**Affiliations:** 1 Centre for Tropical Medicine and Global Health, Nuffield Department of Clinical Medicine, Univeristy of Oxford, Oxford, OX3 7LG, UK; 2 Oxford University Clinical Research Unit, Hanoi, Vietnam; 3 University of Oxford, Siem Reap, 171202, Cambodia; 4 Laos Oxford Mahosot Wellcome Research Unit, Vientiane, Lao People's Democratic Republic; 5 National Hospital for Tropical Diseases, Hanoi, Vietnam; 6 Mahosot Hospital, Vientiane, Lao People's Democratic Republic; 7 Oxford University Clinical Research Unit - Indonesia, Jakarta, Indonesia; 8 Shoklo Malaria Research Unit, Mae Sot, 63110, Thailand; 9 Mahidol-Oxford Tropical Medicine Research Unit, Bangkok, 10400, Thailand; 10 Angkor Hospital for Children, Siem Reap, 171202, Cambodia

**Keywords:** Antimicrobial resistance, Surveillance, Case-based surveillance

## Abstract

**Background:** Case-based surveillance of antimicrobial resistance (AMR) provides more actionable data than isolate- or sample-based surveillance. We developed A Clinically Oriented antimicrobial Resistance surveillance Network (ACORN) as a lightweight but comprehensive platform, in which we combine clinical data collection with diagnostic stewardship, microbiological data collection and visualisation of the linked clinical-microbiology dataset. Data are compatible with WHO GLASS surveillance and can be stratified by syndrome and other metadata. Summary metrics can be visualised and fed back directly for clinical decision-making and to inform local treatment guidelines and national policy.

**Methods:** An ACORN pilot was implemented in three hospitals in Southeast Asia (1 paediatric, 2 general) to collect clinical and microbiological data from patients with community- or hospital-acquired pneumonia, sepsis, or meningitis. The implementation package included tools to capture site and laboratory capacity information, guidelines on diagnostic stewardship, and a web-based data visualisation and analysis platform.

**Results:** Between December 2019 and October 2020, 2294 patients were enrolled with 2464 discrete infection episodes (1786 community-acquired, 518 healthcare-associated and 160 hospital-acquired). Overall, 28-day mortality was 8.7%. Third generation cephalosporin resistance was identified in 54.2% (39/72) of *E. coli* and 38.7% (12/31) of *K. pneumoniae* isolates *.* Almost a quarter of *S. aureus* isolates were methicillin resistant (23.0%, 14/61). 290/2464 episodes could be linked to a pathogen, highlighting the level of enrolment required to achieve an acceptable volume of isolate data. However, the combination with clinical metadata allowed for more nuanced interpretation and immediate feedback of results.

**Conclusions:** ACORN was technically feasible to implement and acceptable at site level. With minor changes from lessons learned during the pilot ACORN is now being scaled up and implemented in 15 hospitals in 9 low- and middle-income countries to generate sufficient case-based data to determine incidence, outcomes, and susceptibility of target pathogens among patients with infectious syndromes.

## Introduction

The benefits of case-based versus isolate-based surveillance of antimicrobial resistance have recently been discussed in peer-reviewed literature. In a recent ACORN protocol manuscript, correspondence on ACORN and a review on surveillance strategies the three purposes of surveillance of antimicrobial resistance (AMR) were listed as “(i) to inform empiric treatment guidelines and clinical decision-making; (ii) to characterize trends in space and time including outbreak detection; and (iii) to provide a benchmark to measure the impact of interventions ( Turner *et al.*, 2020).” It was stated that: “A well-coordinated network of systems, collecting standardised data, that fulfil these purposes provides the evidence base to allow for global comparative analyses and drive strategies for control” ( van Doorn *et al.*, 2020). However, it was also stated that “current AMR surveillance systems are typically passive, pathogen-focused, and based on routine antimicrobial susceptibility testing results generated by clinical microbiology laboratories” ( Lim *et al.*, 2021a; Turner *et al.*, 2020).

The World Health Organization Global Antimicrobial Resistance Surveillance System (WHO GLASS) provides a platform for standardised data collection and submission allowing comparison of data between countries and regions. Sixty-six of 82 enrolled countries (as per 31st July 2019) have submitted data. Most countries submitted isolate or sample-based data, with low- and middle-income countries (LMICs) relatively underrepresented ( World Health Organisation, 2020b). The need for case-based surveillance and its superiority over isolate- or sample-based surveillance was mentioned in the GLASS manual for early implementation ( World Health Organisation, 2015). A recent publication on the burden of AMR in 2019 also mentioned the scarcity of data linking proportions of resistance of bug-drug combinations to clinical syndromes and outcome as a major limitation of data and resulting burden estimates ( Antimicrobial Resistance Collaborators, 2022).

Isolate and sample-based surveillance data without clinical denominators may have various biases favouring resistant organisms due to lack of diagnostic stewardship, underuse of diagnostic microbiology resources and a sampling preference for more severe cases or treatment failures, especially in LMICs ( Lim *et al*., 2021b; Teerawattanasook *et al*., 2017). In addition, many patients are already on antibiotics when sampled as they have access to over-the-counter antibiotics in the community or are transferred with prescribed antibiotics from lower-level clinics. Finally, isolate-based surveillance data also do not inform on impact and cost of AMR, patient, hospital and environmental risk factors or outcomes of drug resistant infections in particular patient groups ( Rempel & Laupland, 2009; Turner *et al*., 2020; van Doorn *et al*., 2020; World Health Organisation, 2016).

Other authors summarised the benefits of case-based surveillance in an opinion piece as: “(i) to allow linking of AMR profiles with patients at risk; (ii) to better inform treatment guidelines; (iii) to identify high-risk populations; (iv) to provide reliable data streams for analyses of effectiveness of interventions; and (v) to study the linkage of AMR phenotypes and burden” ( Ryu *et al*., 2019).

Drawing from these issues and benefits, we have developed ACORN: A Clinically-Oriented antimicrobial Resistance surveillance Network. ACORN aims to be a routine hospital activity of collecting and combining clinical and microbiology data for AMR surveillance with direct feedback and visualisation for end-users. To achieve this, five components are added to a GLASS compatible backbone of microbiology data: (i) active case-finding and provision of diagnostic stewardship materials to support correct use of microbiology diagnostics, (ii) bedside clinical data collection using a short Open Data Kit (ODK) e-questionnaire on a tablet computer, (iii) software solutions to link collected data to existing WHONET-based or other formats of laboratory data to avoid duplication of laboratory data entry, (iv) collection of 28-day mortality data, and (v) direct feedback of local data, visualised using an interactive dashboard. The main objective of ACORN is to generate data in LMICs that can be directly used to inform local treatment guidelines and national policy, in addition to inclusion in international pathogen-focused AMR surveillance systems and burden of disease studies.

Here we describe the results of a pilot in three central hospitals collaborating with the University of Oxford Tropical Network in Southeast Asia: Mahosot Hospital in Vientiane, Laos; Angkor Hospital for Children (AHC) in Siem Reap, Cambodia and the National Hospital for Tropical Diseases (NHTD) in Hanoi, Vietnam. The surveillance protocol was published previously ( Turner *et al*., 2020).

## Methods

### Surveillance design ( Figure 1)

Patients were enrolled through daily active case-finding on 3–5 participating acute admission wards per hospital using clinical diagnosis/suspicion to identify community-acquired infections plus weekly point prevalence surveys for hospital-acquired infections, using case definitions based on those of the European Centres for Disease Control (ECDC; i.e. onset of clinically-suspected infection >48 hours following admission)( European Centre for Disease Prevention and Control, 2016). Patients with clinically suspected meningitis, sepsis or pneumonia were enrolled, with capture of simple severity markers (quick sequential organ failure assessment [qSOFA] for adults and “sepsis six” for children) to enable stratification within syndromes.

**Figure 1.**
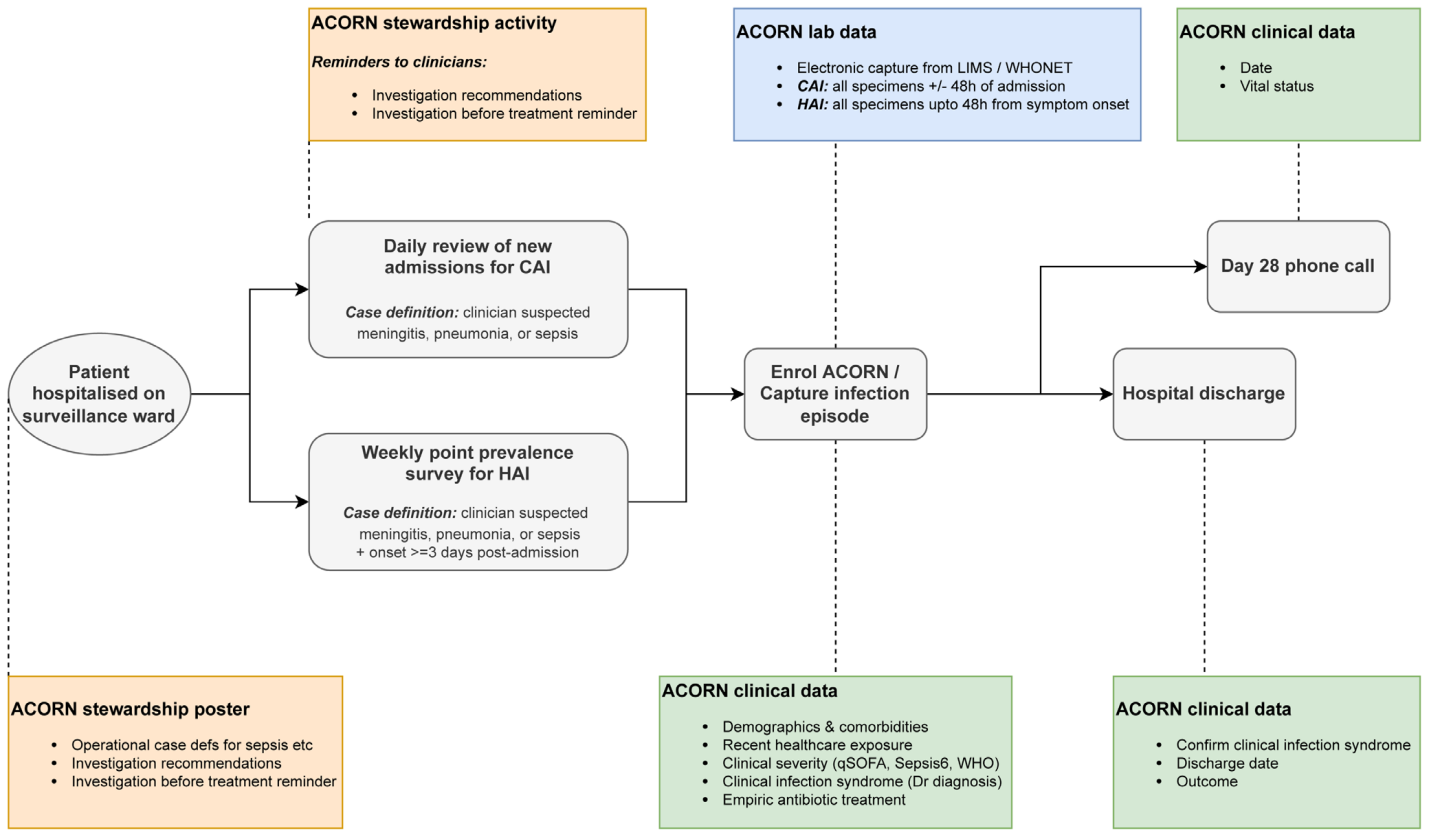
Surveillance design and workflow. CAI: community-acquired infection; HAI: hospital-acquired infection.

Clinical data was captured using password protected Android smartphones / tablets via Open Data Kit (ODK) Collect. Clinical records and electronic hospital information systems of enrolled patients were reviewed by study doctors daily to capture ICD10 codes for infection episode (if recorded), final classification of surveillance diagnosis (confirmed, rejected, updated; for sepsis, the likely source was be captured), hospitalisation outcome (alive, dead, discharged to die at home, left against medical advice) and date and number of days admitted to an intensive care unit. The participant (or parent / legally acceptable representative) was contacted by telephone on day 28 post enrolment to determine health status (alive or dead) and date of death, if appropriate. The protocol implementation package included tools to capture site and laboratory capacity information, guidelines on diagnostic stewardship, and a web-based data visualisation and analysis platform ( Turner *et al*., 2020).

### Site assessments

Prior to study initiation, site and laboratory assessments were conducted on-site to collect general information about the hospital, its patient population, wards ( Table 1), and on the use of clinical microbiology diagnostics and quality management of the laboratory (data not shown).

**Table 1.** ACORN pilot surveillance site summary. ICU: Intensive Care Unit. ID: Infectious Diseases.

	Angkor Hospital for Children	Mahosot Hospital	National Hospital for Tropical Diseases
**Hospital summary**			
Country	Cambodia	Laos	Vietnam
City	Siem Reap	Vientiane	Hanoi
Year founded	1999	1903	2006
Profile	Paediatric	General	Specialist
Level of care	Primary - Tertiary	Primary - Tertiary	National
Subordination	Non-governmental / Charity	Government	Government
Year	2018	2019	2019
Admissions	4013	18392	172955
Transfers in (%)	Unknown	Unknown	67
Beds	82	300	550
Doctors	66	237	130
Nurses	219	400	334
**Surveillance wards**			
Number	3	5	3
Total beds	49	94	168
Details	Paediatric medical 1	Adult ID	Emergency department
	Paediatric medical 2	Paediatric ID	Adult ICU
	Paediatric ICU	General paediatric	Virology & Parasitology
		Adult ICU	
		Paediatric ICU	

### Diagnostic stewardship

Widely used clinical definitions were used in diagnostic stewardship activities (protocol training, poster in doctor’s room, daily ward visits) to support standardisation of clinician diagnosis and specimen collection (see *Extended data,* Supplementary Figures 1 and 2 ( Kestelyn & Van Doorn, 2022)). Surveillance-specific specimen collection was not mandatory; however, we monitored the effectiveness of the diagnostic stewardship materials by tracking the proportion of cases with linked diagnostic specimens.

### Clinical variables

Clinical variable selection was informed by a stakeholder workshop, with representation from WHO GLASS, WHONET, University of Oxford Tropical Network, International Vaccine Institute (IVI), Foundation for Innovative Diagnostics (FIND), Fleming Fund, Centre for Disease Dynamics, Economics & Policy (CDDEP), held in Bangkok in May 2019. Clinical variables in addition to age, sex, ward and date of admission included co-morbidities, date of original admission if transferred from another healthcare facility, hospitalisation and/or surgery in last three months, severity features, presence of medical devices, diagnostic blood culture taken within 24 hours of admission or symptom onset (HAI), systemic antibiotics received in 24 hours before diagnostic blood culture, and empiric antibiotic treatment received. Patients admitted with CAI were re-classified as having healthcare associated infections (HCAI) if they had hospitalisation and/or surgery in last three months. Patients admitted with active infection on transfer from another hospital were considered as a subset of CAI, given that the majority of patients were transferred early and site investigators felt that most transfers were likely escalation of care (Supplementary Figure 3 in the *Extended data*).

### Microbiology data

Locally managed microbiology data, exported from a laboratory information management system (LIMS) or WHONET software, was matched to ACORN clinical data in an RShiny app using patient identifier and specimen collection date. For CAI / HCAI episodes, specimen data from +/- 48 hours of admission date was linked. For HAI episodes, specimen data from up to 48 hours following date of infection onset was linked. Target pathogens for further downstream analyses were the GLASS bloodstream infection relevant species *Streptococcus pneumoniae*, *Staphylococcus aureus*, *Salmonella* spp., *Klebsiella pneumoniae*, *Escherichia coli*, and *Acinetobacter baumannii* but data from all pathogens was captured to permit identification of site-specific pathogens of interest. Raw disk diffusion and minimum inhibitory concentration antimicrobial susceptibility data were interpreted using current (2021) CLSI and EUCAST guidelines ( Clinical and Laboratory Standards Institute, 2021; EUCAST, 2021).

### Ethics and consent

Ethical approval was obtained through the Oxford Tropical Research Ethics Committee (OxTREC 536-19; 21-6-2019), Cambodia National Ethics Committee for Health Research (215-NECHR; 30-8-2019), Laos Ministry of Health – University of Health Sciences Ethics Committee (211/19; 23-9-2019), and NHTD Institutional Review Board, (13/HDDD-NDTU; 18-11-2019). All committees agreed to waive the need for written individual informed consent as this surveillance was defined as a minimal/negligible risk activity, consisting of implementation of accepted quality improvement tools (diagnostic stewardship) and collection and use of limited clinical data that is expected to be collected as part of standard of care. Patients were instead given an information sheet explaining the surveillance project and given the opportunity to opt out on screening and enrolment. No patient samples were collected other than for clinical diagnostic purposes. All patient identifiable data (local hospital identification number, date of birth) were removed during the clinical-laboratory data linkage procedure, rendering an anonymised dataset for analysis and onward sharing.

Preliminary data on key infection syndromes and associated pathogens was collected prior to study start. Challenges and potential solutions to implementation of the surveillance protocol were identified using a baseline AMR knowledge, attitudes, and practices (KAP) survey of clinicians working on the surveillance wards, an open call for feedback, augmented by formal requests for feedback to be presented at investigator meetings (data not shown).

### Sample size and data analysis

There was no formal sample size or target. Wards were chosen based on patient population with preference for those with high proportion of acute admissions from community. The aim was to enrol all eligible and consenting patients admitted to the surveillance wards during the pilot surveillance period. Surveillance data were managed as described above. Tables, graphs, and descriptive statistics were generated using R version 4.1.0 ( R Core Team, 2021). The packages ggplot2 (version 3.3.5) and ComplexUpset (version 1.3.3) were used to plot graphical representations of key results.

## Results

### Admissions and infection episodes

**Figure 2.**
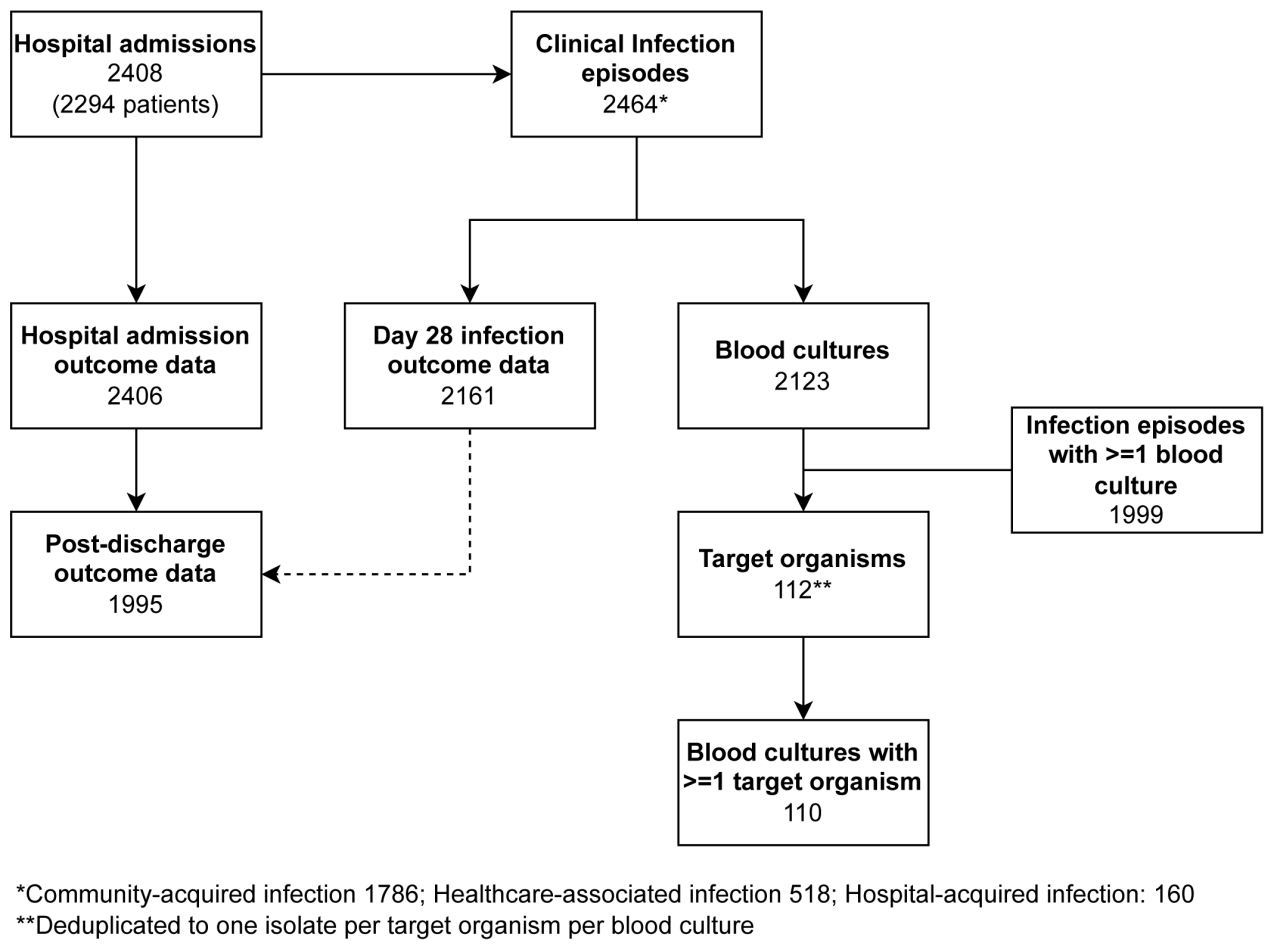
Flowchart of enrolled patients, admissions, episodes and blood cultures.

Patients were enrolled between December 2019 and May 2020, with an extension until end October 2020 after an investigator meeting because of coronavirus disease 2019 (COVID-19) delays and quarantine of the Vietnam site ( Duy *et al*., 2021). ACORN captured data on a total of 2408 patient admissions for 2294 patients, including 2464 discrete infection episodes (1385 AHC, 738 Mahosot, 341 NHTD; median 1, range 1-8 per patient) and 3429 diagnostic specimens. A flowchart is shown in Figure 2 and episodes are shown broken down by month in Supplementary Figure 4 ( *Extended data)*.

The median age at first infection at enrolment was 1.5, 20 and 54 years, with 44.2%, 46.4% and 27.4% female patients at AHC, Mahosot and NHTD, respectively. Among 2464 recorded infection episodes, 2304 were enrolled through daily case-finding with 1786 CAI (72.5%) and 518 classified as HCAI (21.0%). Weekly point-prevalence surveys identified 160 HAI (6.5%). CAI accounted for 66.3%, 88.9% and 62.2% of cases at AHC, Mahosot and NHTD, respectively. At presentation, 253 episodes were clinically diagnosed as meningitis, 677 as pneumonia and 1534 were sepsis. On review at hospital discharge 39.9% (101/253, 1 missing response) meningitis diagnoses, 23.9% (162/677, 2 missing responses) pneumonia diagnoses and 25.7% (395/1534, 4 missing responses) sepsis diagnoses were rejected based on clinical course / diagnostic test results (e.g., radiology) by treating physicians. During the pilot, alternate final diagnoses were not recorded, and thus further results are presented here based on presentation diagnosis.

### Outcomes

**Figure 3.**
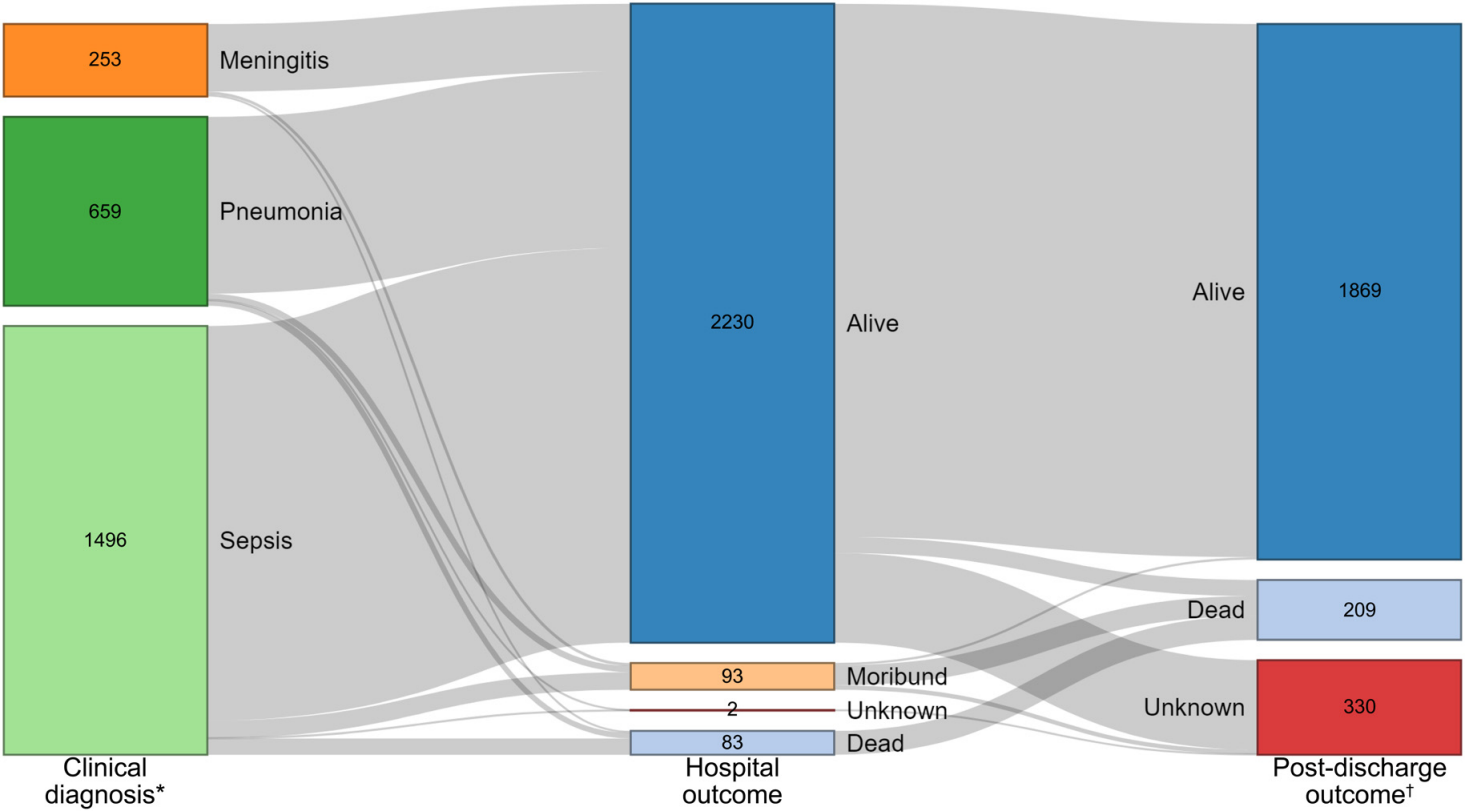
Hospital and post-discharge outcomes for 2408 patient admissions. This plot includes all patient admissions. *Clinical diagnosis denotes first infection captured by surveillance per admission. †Post-discharge outcome denotes 28-day outcome when that time-point occurred following hospital discharge. Unknown post-discharge outcome status reflects either the 28-day outcome occurring before hospital discharge or inability to contact the patient following discharge.

The discharge outcome of 83/2408 (3.5%) admissions was death, with an additional 93 (3.9%) patients discharged moribund. Seven patients had no discharge status recorded. Day-28 follow-up occurred post-discharge for 1995 admissions, at a median of 25 days after discharge (inter-quartile range 21 – 26, range 1 – 56). An additional 126 deaths were identified ( Figure 3). Mortality for adults and children and by syndrome (community-acquired) is shown in Table 2.

**Table 2.** Post discharge outcomes for the total 2408 admissions and per clinical syndrome (community acquired only). Post-discharge outcome denotes 28-day outcome when that time-point occurred following hospital discharge. Unknown post-discharge outcome status reflects either the 28-day outcome occurring before hospital discharge or inability to contact the patient following discharge.

	Alive		Dead		Unknown		Total
**All Admissions**							
Adult	473	66.2%	155	21.7%	86	12.0%	714
Child	1396	82.4%	54	3.2%	244	14.2%	1694
Total	1869	77.6%	209	8.7%	330	13.7%	2408
**Sepsis – community-acquired**							
Adult	231	71.5%	72	22.3%	20	6.2%	323
Child	657	83.6%	22	2.8%	107	13.6%	786
Total	888	80.1%	94	8.5%	127	11.5%	1109
**Pneumonia – community-acquired**							
Adult	94	69.6%	26	19.3%	15	11.1%	135
Child	305	88.7%	3	0.9%	36	10.5%	344
total	399	83.3%	29	6.1%	51	10.6%	479
**Meningitis – community-acquired**							
Adult	43	64.1%	8	11.9%	16	23.9%	67
Child	120	91.6%	4	3.1%	7	5.3%	131
Total	163	82.3%	12	6.1%	23	11.6%	198

Restricting to 2287 first infection episodes where discharge status was available (i.e., each enrolled patient represented as a single infection episode and admission), 2114 (92.4%) patients were discharged alive and expected to recover with 92 patients (4.0%) discharged moribund and 81 (3.5%) whose hospital outcome was death. There were no clear differences between hospital outcome by clinical syndrome. However, a higher proportion of patients with HAI were discharged moribund (11.6%, 11/95) compared to those with CAI (3.2%, 57/1567) or HCAI (23/496, 4.6%) (Supplementary Table 1, *Extended data*).

### Blood cultures

Data from 2123 blood cultures was captured. At least one blood culture was taken in 81.1% (1999/2464) of infection episodes in total and in 87.7% (1215), 74.8% (552) and 68.0% (232) of episodes at AHC, Mahosot and NHTD, respectively. Collected blood cultures are summarised by syndrome and origin of infection in Table 3. Where data were available (1644/1999 episodes), there was no history of antibiotics in the 24 hours preceding sample collection in 78.0% episodes (overall 1283/1644; AHC 722/903 [80.0%]; Mahosot 437/518 [84.4%]; NHTD 124/223 [55.6%]). Positive blood culture results were obtained from 75/1283 (5.8%) patients who had not received antibiotics, versus 13/361 (3.6%) of patients who had received antibiotics.

**Table 3.** Blood culture collection summary for 2464 infection episodes, stratified by location, patient age group, diagnosis, and infection category. CAI: community-acquired infection; HCAI: healthcare-associated infection; HAI: hospital-acquired infection.

At least one blood culture collected, n (%)	AHC		Mahosot		NHTD	
	Adult	Child	Adult	Child	Adult	Child
**Diagnosis**						
Meningitis	-	112 / 130 (86.2%)	30 / 30 (100.0%)	27 / 29 (93.1%)	48 / 61 (78.7%)	3 / 3 (100.0%)
Pneumonia	-	283 / 376 (75.3%)	55 / 83 (66.3%)	40 / 112 (35.7%)	64 / 102 (62.7%)	3 / 4 (75.0%)
Sepsis	-	820 / 879 (93.3%)	235 / 276 (85.1%)	165 / 208 (79.3%)	111 / 165 (67.3%)	3 / 6 (50.0%)
**Infection timing**						
CAI	-	800 / 918 (87.1%)	270 / 321 (84.1%)	221 / 335 (66.0%)	153 / 204 (75.0%)	5 / 8 (62.5%)
HCAI	-	316 / 358 (88.3%)	46 / 63 (73.0%)	10 / 13 (76.9%)	48 / 79 (60.8%)	4 / 5 (80.0%)
HAI	-	99 / 109 (90.8%)	4 / 5 (80.0%)	1 /1 (100.0%)	22 / 45 (48.9%)	-

Blood cultures were reported to contain contaminants (coagulase negative staphylococci, micrococci or Gram-positive bacilli) in 70/1310 (5.3%) and 31/569 (5.4%) specimens at AHC and Mahosot, respectively. At NHTD these numbers were not available as the laboratory does not report growth of these contaminants back to the wards, often after checking with treating physicians.

**Figure 4.**
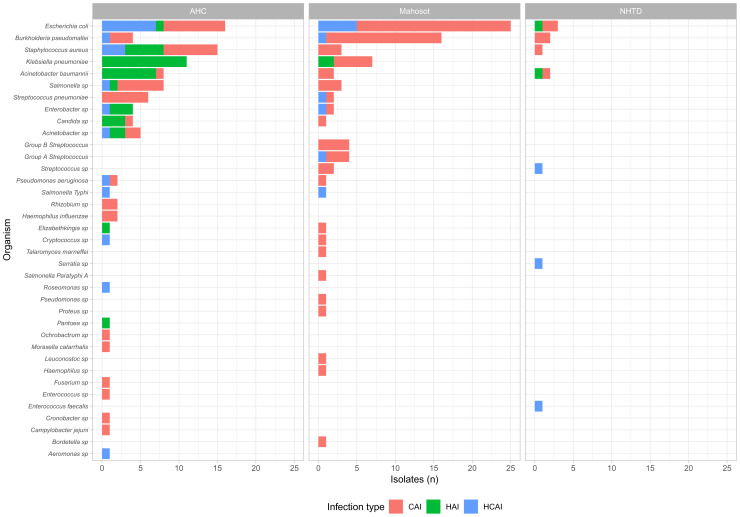
Potential pathogens isolated from 2123 blood cultures, by location and timing of infection onset. CAI: community-acquired infection; HCAI: healthcare-associated infection; HAI: hospital-acquired infection.

In total, 192 potentially pathogenic organisms were isolated from 2123 blood cultures from 1999 infection episodes. Six out of seven of the most frequently isolated organisms were target organisms (115/192): 44 *E. coli*, 19 *S. aureus*, 18 *K. pneumoniae*, 12 *A. baumannii* (and an additional 5 *Acinetobacter* spp.), 14 *Salmonella* spp. (including 2 *S.* Typhi and 1 *S.* Paratyphi A), and 8 *S. pneumoniae.* At least one target organism was cultured from 110/2123 (5.2%) blood cultures. In addition, *Burkholderia pseudomallei,* a regionally important pathogen, was isolated from 22 blood cultures ( Figure 4, Supplementary Table 2, *Extended data*).

Among 1999 blood-cultured infection episodes, the incidence of blood stream infections (BSI) caused by any target organism was 51 per 1000 episodes (95% confidence interval [CI] 42 – 63): 18 (95% CI 5 – 47) for meningitis, 25 (95% CI 12 – 44) for pneumonia and 66 (95% CI 53 – 81) for sepsis. Breakdowns of BSI incidence by target organism, age group and infection timing are shown in Supplementary Tables 3 – 4 ( *Extended data)*.

The proportion of blood cultured infection episodes rendering a target organism was positively correlated with qSOFA score (17% [9/53] of score 3, 3% [3/102] of score 0) and to a lesser extent to the number of “Sepsis 6” severity indicators in children ( Table 4). Both among children and adults the likelihood of culturing a target organism was higher among HAI than CAI and HCAI episodes ( Table 5).

**Table 4.** Bloodstream infection detection by clinical severity and age group.

Age group	Severity score *	Episode with target organism BSI (n)	Episodes with blood culture collected (n)	BSI (%)
**Adults**				
qSOFA	0	3	102	2.9
	1	7	201	3.5
	2	20	187	10.7
	3	9	53	17.0
**Child**				
Sepsis6*	0	5	359	1.4
	1	19	444	4.3
	2	25	394	6.3
	3	8	148	5.4
	4	7	111	6.3

*Sepsis6 is not a score per se, so the number of indicator items detected is summarised

**Table 5.** Bloodstream infection detection by timing of infection onset and age group. CAI: community-acquired infection; HCAI: healthcare-associated infection; HAI: hospital-acquired infection.

Age group	Infection category	Episode with target organism BSI (n)	Episodes with blood culture collected (n)	BSI (%)
**Adult**	CAI	30	423	7.1
	HCAI	6	94	6.4
	HAI	3	26	11.5
**Child**	CAI	34	1026	3.3
	HCAI	11	330	3.3
	HAI	19	100	19.0

### Non-blood cultures

Data from 1306 non-blood culture specimens was captured. Cerebrospinal fluid (CSF) specimen data was captured in 58% of meningitis episodes in total and in 58% (76), 56% (33) and 58% (37) of episodes at AHC, Mahosot and NHTD, respectively. A total of 147 CSF specimens were captured from patients with clinically suspected meningitis, 14 were culture positive yielding a single potentially pathogenic isolate in each case (Supplementary Table 5, *Extended data*). Lower respiratory tract specimen data was captured in 6% of pneumonia episodes in total and in 4% (14), 6% (3) and 19% (20) of episodes at AHC, Mahosot and NHTD, respectively. Overall, 41 lower respiratory tract specimens were captured from patients with clinically suspected pneumonia, more frequently from hospital-acquired (9/42, 21.4%) than community-acquired (26/479, 5.4%) or healthcare-associated (5/156, 3.2%) pneumonia episodes. Of these, 13 were culture positive and 26 potentially pathogenic isolates were recovered (Supplementary Table 6, *Extended data*). The range of non-blood culture specimen and organism data are summarized in Supplementary Table 7 and Supplementary Figure 5 ( *Extended data*).

### Antimicrobial susceptibility test results

**Figure 5.**
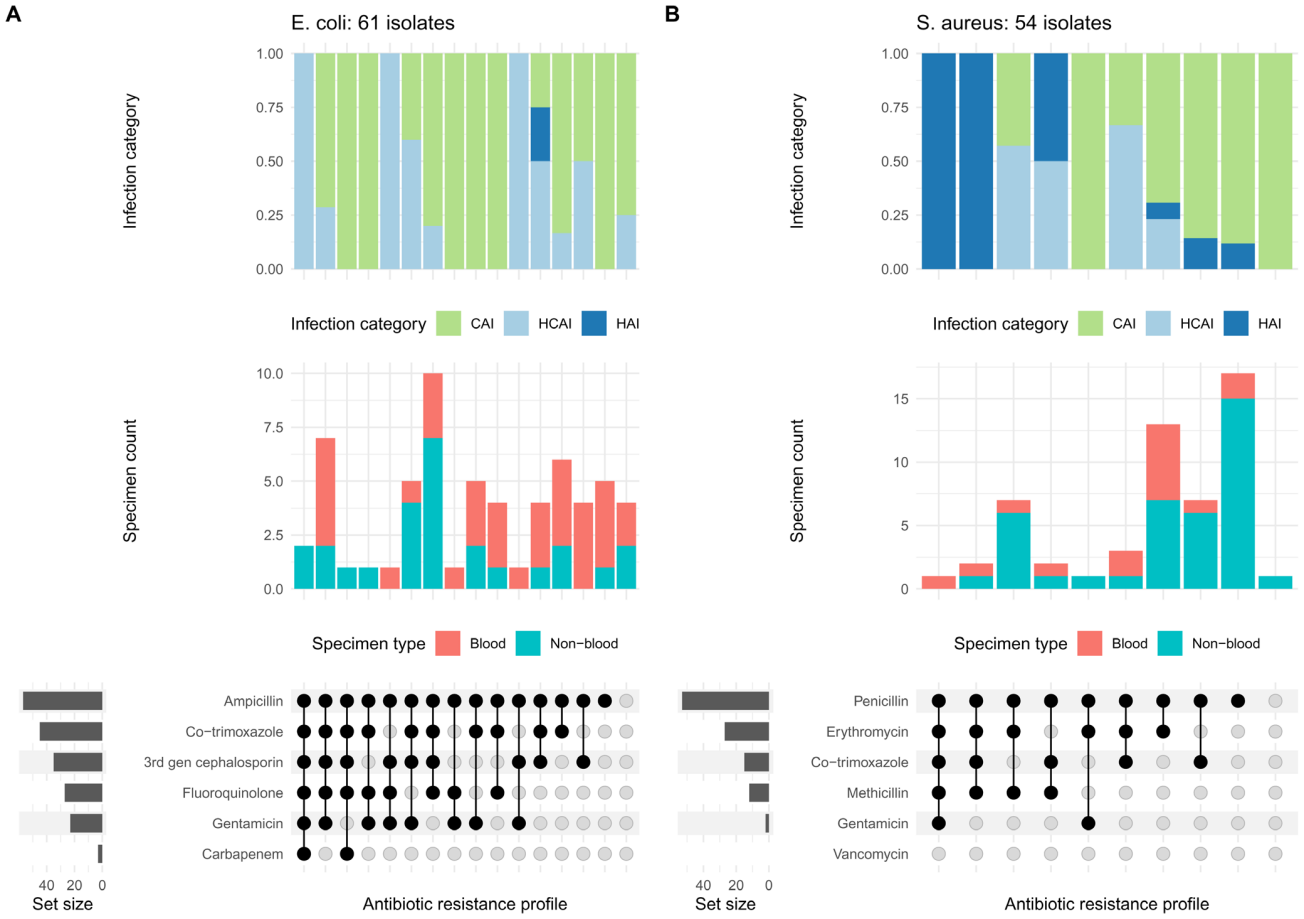
Antimicrobial susceptibility profiles for ( **A**) 61 *Escherichia coli* isolates and ( **B**) 54 *Staphylococcus aureus* isolates (all site data combined). The first isolate per specimen type for each patient was included. In each panel, the top plot summarises the infection category from which isolates were obtained, as a proportion (CAI, community-acquired; HCAI, healthcare-associated; HAI, hospital-acquired); the middle bar plot indicates the specimen (and thus isolate) count coloured by specimen type; and the bottom plot shows the antimicrobial susceptibility profile for the isolates (black filled circles indicate resistance and grey filled circles indicate susceptibility). The horizontal bars (“Set size”), indicate the number of isolates testing resistant a single antibiotic / antibiotic class (e.g., top row for *S. aureus* is penicillin and almost all isolates are resistant).

Due to the small numbers of isolates generated in the pilot, detailed exploration of the data was considered inappropriate. However, examples of AST profiles were generated for the two most prevalent organisms, *E. coli* and *S. aureus* (61/80 and 54/66 isolates, first isolate per specimen type per patient with all core antibiotics tested; Figure 5A/B). Susceptibility to key antibiotic classes is summarised in Supplementary 8 ( *Extended data*). After deduplication to the first isolate per species per patient, there were >30 isolates for *E. coli, K. pneumoniae,* and *S. aureus.* Third generation cephalosporin resistance was identified in 54.2% (39/72) of *E. coli* and 38.7% (12/31) of *K. pneumoniae* isolates *.* Almost a quarter of *S. aureus* isolates were methicillin resistant (123.0%, 4/61).

## Discussion

To overcome the biases of isolate-based surveillance and improve direct feedback of AMR surveillance data to hospitals and prescribing doctors we developed ACORN. ACORN starts with a patient rather than a pathogen, and consists of bedside collection of clinical metadata that is subsequently linked to clinical microbiology laboratory data and displayed on an online or offline dashboard. ACORN was piloted in three hospitals in Southeast Asia between December 2019 and October 2020.

We show objectives of the pilot with predefined outcome measures and how we met those in Table 6. Before and after informal satisfaction surveys among clinical and laboratory staff, and discussions at investigator meetings, showed that overall, ACORN was acceptable and feasible to implement with the provided per patient compensation for study doctors / nurses for their time and presence of site coordinators on the wards. Doctors in general appreciated the results that were generated and how they were displayed. Feedback on specific components was used to update enrolment criteria and other study procedures, discussed further below.

**Table 6.** Objectives, formulated outcome measures and how these were met.

Objectives	Outcome Measures	Result
**Primary:** To develop, implement and assess a hospital-based system for patient-centred surveillance of Drug Resistant Infections	A protocol and guideline for implementation of this system for further roll-out in other sites	Feedback was collected during the pilot and on post pilot assessment and was used to update the protocol
**Secondary:** To systematically characterize drug-resistant infections based on important clinical syndromes, to adequately inform treatment guidelines To implement clinical syndrome-guided diagnostic stewardship of patients with suspected infection To determine the duration, cost of hospitalisation and patient outcome of DRI and non-DRI	Antimicrobial susceptibility data with both pathogen and clinical denominators, including predefined subgroups Proportion of timely and correctly sampled patients per syndrome Antimicrobial susceptibility data, cost estimates, 28-day mortality data	Collection and combination of clinical and microbiological data was feasible (all secondary objectives), subgroup analysis showed clear differences by antibiotic use and origin of infection Data were collected and used for real-time feedback and further development of protocol Data were collected and shown
**Tertiary:** To evaluate the feasibility and acceptability of the surveillance system and package of tools	Results of clinician and laboratory technician surveys	Feedback was collected using before and after surveys and results were used to update the protocol

Denominators of generated data and antibiograms in this pilot were too small for immediate use in clinical guidelines and definitive analyses of AMR impacts. However, the framework developed, and iterated as described below, will permit such usage during longer periods of surveillance.

Data from 2464 clinical infection episodes were collected and matched with 192 potentially pathogenic isolates from blood culture and an additional 229 potentially pathogenic isolates from other specimens. In total, 290 infection episodes (11.8%) were matched to a pathogen. Receiving antibiotics when blood culture was taken was associated with a lower proportion of positive culture results (3.6 vs. 5.8%).

In total, 83 patients died in hospital and an additional 126 deaths occurred following hospital discharge (overall mortality 8.7% [209/2408]). Of these, 93 patients were discharged moribund to die at home rather than the hospital, which is often preferred by patients and families in the cultural contexts of these three countries ( Tran *et al*., 2018). When broken down by age, mortality among adults was consistently higher: 21.7% among adults vs. 3.2% among children in total, and 22.3%, 19.3%, 11.9% among adults vs 2.8%, 0.9%, 3.1% among children for sepsis, pneumonia and meningitis, respectively. These percentages are very similar to the estimates from our previous sepsis study in the region ( Southeast Asia Infectious Disease Clinical Research Network, 2017). 

Low numbers of lower respiratory tract samples were collected for patients with a clinical diagnosis of pneumonia, both among children and adults. Hospital-acquired pneumonia cases were sampled more frequently than community- or healthcare-associated pneumonia cases and this perhaps reflects views on clinical utility and the fact that around 50% of hospital-acquired pneumonia patients were mechanically ventilated. High quality sputum specimens from community-acquired pneumonia cases are notoriously hard to generate, resulting in culture results that frequently represent upper respiratory tract colonisation.

These results show that case-based surveillance of AMR requires collection of data from a considerably larger number of patients to achieve the same amount of isolate based information compared to strictly isolate based surveillance. However, the clinical data collected is richer and allows for stratification by syndrome, severity, origin of infection and other metadata allowing for rapid actionable feedback to clinicians and hospitals. The results from this pilot also show that ACORN is feasible to deliver, but at this stage and in these settings required dedicated clinical and data staff (time required for clinical data collection and processing / matching of data on site and centrally) and therefore funding was a prerequisite for delivery. During further roll-out we will assess to what extent ACORN surveillance can become part of routine work on site and self-sustainable.

Categorisation between CAI, HCAI and HAI was heterogeneous between three sites, reflecting differences in patient populations. Pragmatically, we disregarded transfers from lower-level hospitals because most were hospitalised <48h before transfer and this was considered escalation of care and transferred cases were classified as CAI. This may have led to some HAIs being misclassified, especially at NHTD which receives >70% of its patients from peripheral sites. Detecting HAI with weekly point-prevalence surveys but without case definitions may not have caught all infections. Conversely, some *Salmonella* and *B. pseudomallei* infections were labelled as HAI/HCAI according to protocol, which probably does not reflect their true origin of infection. Similarly, the proportion of patients with blood culture taken per site and syndrome and the proportions of rejected admission diagnoses were heterogeneous between sites, showing rigorous on-site diagnostic stewardship and clear guidance regarding case definitions is important. Clinical feedback during the pilot highlighted that several patients with a pathogenic organism grown from blood were not included in surveillance due to lack of a clearly documented clinical diagnosis. Alternative enrolment strategies were thus considered and a two-week audit of patient numbers on surveillance wards was undertaken (data not shown). We concluded that enrolment could be improved by selection of patients based on clinician antibiotic treatment choices, rather than written diagnosis.

Phase 2 of ACORN is currently being implemented, with a wider roll-out in 15-18 sites in 9 countries in Africa and Asia. After having reviewed the pilot experiences with study staff from hospitals and research units, the following changes to the protocol were made:

- Pilot enrolment criteria were based on clinical diagnosis without requirement for specific criteria to be checked. Diagnoses could be confirmed or rejected at discharge. For ACORN2 the only requirement for patient enrolment will be the presence of a clinically suspected acute infection and the intention to commence / receipt of intravenous antibiotics on screening for eligibility. Patients will be categorised subsequently by syndrome on discharge.
- An adapted pragmatic clinical syndrome list modified from the Global PPS protocol and the WHO attributable mortality protocol will be used ( World Health Organisation, 2020a). Study staff can choose the most appropriate clinical syndrome without the need for checking specific case definition boxes.
- We will convene meetings with surveillance experts to discuss pragmatic syndromic definitions of community and hospital acquired infections for use in future generations of ACORN.
- Data capture on day 28 will be more nuanced and expanded to allow for more detailed capture of ongoing morbidity and health economics analyses.

While the ACORN pilot and ACORN2 collect data in a manner that is compatible with GLASS AMR surveillance, in ACORN2 there will be a direct link with the WHO GLASS team and alignment of data capture for *E. coli* and *S. aureus* bloodstream infections with the WHO attributable mortality protocol ( World Health Organisation, 2020a).

To enhance data utilisation, linkages have been made with a range of investigators internationally to implement ACORN or ACORN like AMR surveillance, or reuse ACORN data, in projects where innovative data collection methods are employed such as Crit Care Asia, ADVANCE-ID, and other ongoing multi-country projects ( Crit Care Asia, 2020; Walker *et al*., 2021).

## Conclusion

ACORN was feasible and generated site-usable data in a pilot phase. Lessons were learned and are being implemented in ACORN2, which will generate sufficient data to determine the incidence of GLASS target pathogen blood stream infection in range of LMIC clinical settings. Additional analyses will determine the impact of AMR on clinical outcomes and healthcare costs in such settings.

## Data Availability

Individual participant data collected for the pilot phase of ACORN will not be made available to third parties at this stage of the devlopment of ACORN. ACORN pilot data were used internally for evaluation and proof of principle purposes, and were only presented descriptively here and not used to generate or prove hypotheses. Further downstream iterations of ACORN will have data sharing as a priority, as ultimately the purpose of ACORN is to create a better global database of clinical and laboratory AMR data. Data dictionaries, study protocol, informed consent forms and other study documents are available on publication at https://acornamr.net. Should reviewers or readers wish to access individual participant data, please contact the corresponding author at rvandoorn@oucru.org. We will work with our internal data access committee and the institutional review boards of the sites to get the appropriate approvals for sharing of suffciciently anonymised data. Oxford University Research Archive: ACORN (A Clinically Oriented antimicrobial Resistance Network) supplementary material. https://doi.org/10.5287/bodleian:xq89Qnvke ( Kestelyn & Van Doorn, 2022). This project contains the following extended data: Supplementary figures Supplementary tables Data are available under the terms of the Creative Commons Attribution 4.0 International license (CC-BY 4.0).

## References

[ref-1] Antimicrobial Resistance Collaborators: Global burden of bacterial antimicrobial resistance in 2019: a systematic analysis. *Lancet.* 2022;399(10325):629–55. 10.1016/S0140-6736(21)02724-0 35065702 PMC8841637

[ref-2] Clinical and Laboratory Standards Institute: Performance Standards for Antimicrobial Susceptibility Testing M100-S31.CLSI;2021. Reference Source 10.1128/JCM.00213-21 PMC8601225 34550809

[ref-3] Crit Care Asia: Establishing a critical care network in Asia to improve care for critically ill patients in low- and middle-income countries. *Crit Care.* 2020;24(1):608. 10.1186/s13054-020-03321-7 33059761 PMC7558669

[ref-4] Duy C Nong VM Van Ngo A : Nosocomial Coronavirus Disease Outbreak Containment, Hanoi, Vietnam, March-April 2020. *Emerg Infect Dis.* 2021;27(1):10–17. 10.3201/eid2701.202656 33207153 PMC7774565

[ref-6] European Centre for Disease Prevention and Control: Point Prevalence Survey of Healthcare Associated Infections and Antimicrobial Use in European Acute Care Hospitals—Protocol Version 5.3.ECDC;2016. Reference Source

[ref-5] European Committee on Antimicrobial Susceptibility Testing (EUCAST): Breakpoint tables for interpretation of MICs and zone diameters.V11.0;2021; [Accessed 18-08-2022]. Reference Source

[ref-7] Kestelyn E Van Doorn R : ACORN (A Clinically Oriented Antimicrobial Resistance Network) Supplementary Material.University of Oxford, [Dataset],2022.

[ref-8] Lim C Ashley EA Hamers RL : Surveillance strategies using routine microbiology for antimicrobial resistance in low- and middle-income countries. *Clin Microbiol Infect.* 2021a;27(10):1391–9. 10.1016/j.cmi.2021.05.037 34111583 PMC7613529

[ref-9] Lim C Hantrakun V Teerawattanasook N : Impact of low blood culture usage on rates of antimicrobial resistance. *J Infect.* 2021b;82(3):355–62. 10.1016/j.jinf.2020.10.040 33278401 PMC7994019

[ref-10] R Core Team: R: A language and environment for statistical computing.Vienna, Austria: R Foundation for Statistical Computing.;2021.

[ref-11] Rempel OR Laupland KB : Surveillance for antimicrobial resistant organisms: potential sources and magnitude of bias. *Epidemiol Infect.* 2009;137(12):1665–73. 10.1017/S0950268809990100 19493372

[ref-12] Ryu S Cowling BJ Wu P : Case-based surveillance of antimicrobial resistance with full susceptibility profiles. *JAC Antimicrob Resist.* 2019;1(3):dlz070. 10.1093/jacamr/dlz070 32280945 PMC7134534

[ref-13] Southeast Asia Infectious Disease Clinical Research Network: Causes and outcomes of sepsis in southeast Asia: a multinational multicentre cross-sectional study. *Lancet Glob Health.* 2017;5(2):e157–e67. 10.1016/S2214-109X(17)30007-4 28104185 PMC5332551

[ref-14] Teerawattanasook N Tauran PM Teparrukkul P : Capacity and Utilization of Blood Culture in Two Referral Hospitals in Indonesia and Thailand. *Am J Trop Med Hyg.* 2017;97(4):1257–61. 10.4269/ajtmh.17-0193 28722626 PMC5637610

[ref-15] Tran HT Nguyen HP Walker SM : Validation of verbal autopsy methods using hospital medical records: a case study in Vietnam. *BMC Med Res Methodol.* 2018;18(1):43. 10.1186/s12874-018-0497-7 29776431 PMC5960129

[ref-16] Turner P Ashley EA Celhay OJ : ACORN (A Clinically-Oriented Antimicrobial Resistance Surveillance Network): a pilot protocol for case based antimicrobial resistance surveillance [version 2; peer review: 4 approved]. *Wellcome Open Res.* 2020;5:13. 10.12688/wellcomeopenres.15681.2 32509968 PMC7250055

[ref-17] van Doorn HR Ashley EA Turner P : Case-based surveillance of antimicrobial resistance in the ACORN (A Clinically Oriented Antimicrobial Resistance Surveillance Network) study. *JAC Antimicrob Resist.* 2020;2(1):dlaa018. 10.1093/jacamr/dlaa018 32280946 PMC7134533

[ref-18] Walker AS White IR Turner RM : Personalised randomised controlled trial designs-a new paradigm to define optimal treatments for carbapenem-resistant infections. *Lancet Infect Dis.* 2021;21(6):e175–e81. 10.1016/S1473-3099(20)30791-X 33894130 PMC7614698

[ref-19] World Health Organisation: Global Antimicrobial Resistance Surveillance System - Manual for Early Implementation.Geneva: World Health Organisation;2015. Reference Source

[ref-20] World Health Organisation: Global AMR Surveillance System: Diagnostic stewardship: A guide to implementation in antimicrobial resistance surveillance sites.Geneva: World Health Organisation;2016. Reference Source

[ref-21] World Health Organisation: GLASS method for estimating attributable mortality of antimicrobial resistant bloodstream infections.Geneva: World Health Organisation,2020a. Reference Source

[ref-22] World Health Organisation: Global Antimicrobial Resistance and Use Surveillance System (GLASS) Report Early implementation 2020.Geneva: World Health Organisation;2020b. Reference Source

