# Supplementary material for: A Clinically Oriented antimicrobial Resistance surveillance Network (ACORN): pilot implementation in three countries in Southeast Asia, 2019-2020
Source: Wellcome Open Res. 2022 Dec 22;7:309. [Version 1] doi: 10.12688/wellcomeopenres.18317.1 (PMC10579863; doi:10.12688/wellcomeopenres.18317.1)
Supplement: Peer review history

# Reviewer report: Reviewer response for version 1

**DOI:** 10.21956/wellcomeopenres.20302.r65293

**Published:** 2023-10-16

**Authors:** Dickson Aruhomukama

**Affiliations:** 1 Department of Medical Microbiology, College of Health Sciences, Makerere University, Kampala, Central Region, Uganda

I had the privilege of reviewing the ACORN II protocol, which serves as a continuation of this research, even before being proposed as the reviewer for this specific manuscript. I have observed that several changes have been implemented in the subsequent study (ACORN II), influenced by the insights from this initial study (which can be seen as the pilot for ACORN II). Due to this, I find myself more at ease with this version of the manuscript.

Is the work clearly and accurately presented and does it cite the current literature?

Partly

If applicable, is the statistical analysis and its interpretation appropriate?

Partly

Are all the source data underlying the results available to ensure full reproducibility?

Partly

Is the study design appropriate and is the work technically sound?

Partly

Are the conclusions drawn adequately supported by the results?

Partly

Are sufficient details of methods and analysis provided to allow replication by others?

Partly

Reviewer Expertise:

Clinical Microbiology, Computational Microbiology,  Clinical Bioinformatics (Genomics)

I confirm that I have read this submission and believe that I have an appropriate level of expertise to confirm that it is of an acceptable scientific standard.

# Reviewer report: Reviewer response for version 1

**DOI:** 10.21956/wellcomeopenres.20302.r53853

**Published:** 2023-02-21

**Authors:** Frank van Leth

**Affiliations:** 1 Department of Health Sciences, Faculty of Science, Vrije Universiteit, Amsterdam, The Netherlands

The study by van Doorn et al. describes the result of the pilot phase of the ACORN project, which has the objective to provide case-based AMR surveillance. Using the inpatient setting, the project matches clinical information, laboratory data, and outcome data from individual patients admitted at an emergency ward for suspected infections. Case-based AMR surveillance should overcome the inherent bias seen in conventional isolate- or sample-based AMR surveillance, and as such contribute better to guidance of antibiotic therapy. The authors conclude that the implementation of such a surveillance strategy is feasible and decided to scale up the project to 15-18 hospitals in the region.

 The manuscript provides detailed descriptive information on the entire process from enrolment to assessing outcome data. By doing so, the authors show that the approach can lead to a rich dataset that provides insight in diagnostic stewardship, clinical record keeping, and follow-up procedures. As such, the manuscript delivers clearly on its objectives

**Main comment.**

 There is no doubt that current surveillance is severely hampered by the approach taken. The WHO GLASS initiative is welcome but can only provide a limited view of the AMR-situation, given its sample-based approach. Case-based surveillance is urgently needed, something acknowledged by WHO, and being addressed by the ACORN project.

 It is therefore somewhat surprising that the Discussion section does not further elaborate how case-based AMR  surveillance could be designed. Adding clinical data to samples submitted to the laboratory is one option, especially if this is done through active case-finding and point-prevalence surveys as in ACORN. But does this inform antibiotic use in those large number of patients not being admitted to an emergency ward? The majority of antibiotic prescription is done in an outpatient setting. The discussion section would be strengthened if the potential limitation of the inpatient setting of ACORN is discussed.

 Furthermore, there are other approaches to case-based AMR surveillance being discussed in the literature. Some of these try to overcome one of the limitations of ACORN (as mentioned in the Discussion section): the need for the large number of patients to be able to decipher AMR-profiles with a reasonable level of precision. Without these profiles, guidance on (empirical) antibiotic treatment will be impossible.

**Minor comments**

 In Table 6, the authors mention the coverage of the objective of cost assessment. I did not see this in the main text, nor in the supplemental material.

 In Figure 5 middle bar chart, the Y-axis is labelled in steps of 0.5. As it refers to the number of isolates, I suggest to change this to make assessment easier.

 As direct visualisation is a major objective of the project, it would be great to see an example. The shiny-app does not show anything as no data are uploaded. Could there be a screen-shot of how an actual visualisation on the dashboard would look like?

Is the work clearly and accurately presented and does it cite the current literature?

Yes

If applicable, is the statistical analysis and its interpretation appropriate?

Not applicable

Are all the source data underlying the results available to ensure full reproducibility?

Partly

Is the study design appropriate and is the work technically sound?

Yes

Are the conclusions drawn adequately supported by the results?

Yes

Are sufficient details of methods and analysis provided to allow replication by others?

Yes

Reviewer Expertise:

Infectious disease epidemiologist working in the field of antimicrobial resistance surveillance

I confirm that I have read this submission and believe that I have an appropriate level of expertise to confirm that it is of an acceptable scientific standard.
